# A mutation in the H/ACA box of telomerase RNA component gene (*TERC*) in a young patient with myelodysplastic syndrome

**DOI:** 10.1186/1471-2350-15-68

**Published:** 2014-06-19

**Authors:** Yasutaka Ueda, Rodrigo T Calado, Anna Norberg, Sachiko Kajigaya, Göran Roos, Eva Hellstrom-Lindberg, Neal S Young

**Affiliations:** 1 Hematology Branch, National Heart, Lung, and Blood Institute, National Institutes of Health, Bldg 10-CRC, Rm 3E-5216, 9000 Rockville Pike, Bethesda, MD 20892, USA; 2 Department of Internal Medicine, University of São Paulo at Ribeirão Preto Medical School, Ribeirão Preto, São Paulo, Brazil; 3 Department of Medical Biosciences, Medical and Clinical Genetics, Umeå University, Umeå, Sweden; 4 Department of Medical Biosciences, Pathology, Umeå University, Umeå, Sweden; 5 Karolinska University Hospital and Karolinska Institute, Stockholm, Sweden

**Keywords:** Myelodysplastic syndrome (MDS), Telomerase RNA component (TERC), H/ACA box, Southern blotting, Single Telomere Elongation Length Analysis (STELA), RNA fluorescence in situ hybridization (RNA FISH)

## Abstract

**Background:**

Telomeres are repeated sequences (the hexanucleotide TTAGGG in vertebrates) located at chromosome ends of eukaryotes, protecting DNA from end joining or degradation. Telomeres become shorter with each cell cycle, but telomerase, a ribonucleoprotein complex, alleviates this attrition. The telomerase RNA component (TERC) is an essential element of telomerase, serving as a template for telomere elongation. The H/ACA domain of TERC is indispensable for telomere biogenesis. Mutations in the telomerase components allow accelerated telomere loss, resulting in various disease manifestations, including bone marrow failure. To date, this is the first detailed report of an H-box mutation in TERC that is related to human disease.

**Case presentation:**

A 26-year-old man with myelodysplastic syndrome (MDS) had very short telomeres. Sequencing identified a single heterozygous mutation in the H box of the patient’s *TERC* gene. The same mutation was also present in his father and his son, demonstrating that it was germline in origin. The telomere length in the father’s blood was shorter compared to age-matched healthy controls, while it was normal in the son and also in the sperm cells of the patient. *In vitro* experiments suggested that the mutation was responsible for the telomere shortening in the patient’s leukocytes and contributed to the pathogenesis of bone marrow failure in our patient.

**Conclusion:**

We analyzed a mutation (A377G) in the H box of *TERC* in a young MDS patient who had significantly short-for-age telomeres. As telomeres protect chromosomes from instability, it is highly plausible that this genetic lesion was responsible for the patient’s hematological manifestations, including marrow failure and aneuploidy in the hematopoietic stem cell compartment.

## Background

Telomeres are composed of a highly conserved repetitive DNA sequence (TTAGGG) at the ends of eukaryotic chromosomes with associated proteins, collectively termed shelterin. Telomeres protect DNA from fusion with neighboring chromosomes or degradation by exonuclease (reviewed in [1]). In humans, telomeres are 9 – 15 kb in length, and 100–200 base pairs of telomeric sequence are lost in each cell replication [2]. The molecular basis of loss is inability to fully replicate DNA of the lagging strand during replication (known as the end replication problem) [3]. When telomere length is critically short, cells enter replicative senescence or die [4]. Telomere shortening is alleviated by enzymatic activity of telomerase, a complex composed of telomerase reverse transcriptase (TERT), telomerase RNA component (TERC), and other proteins (reviewed in [1]). In most human somatic cells, telomerase activity is suppressed; however, telomerase is active in proliferating progenitor cells.

**Figure 1:**
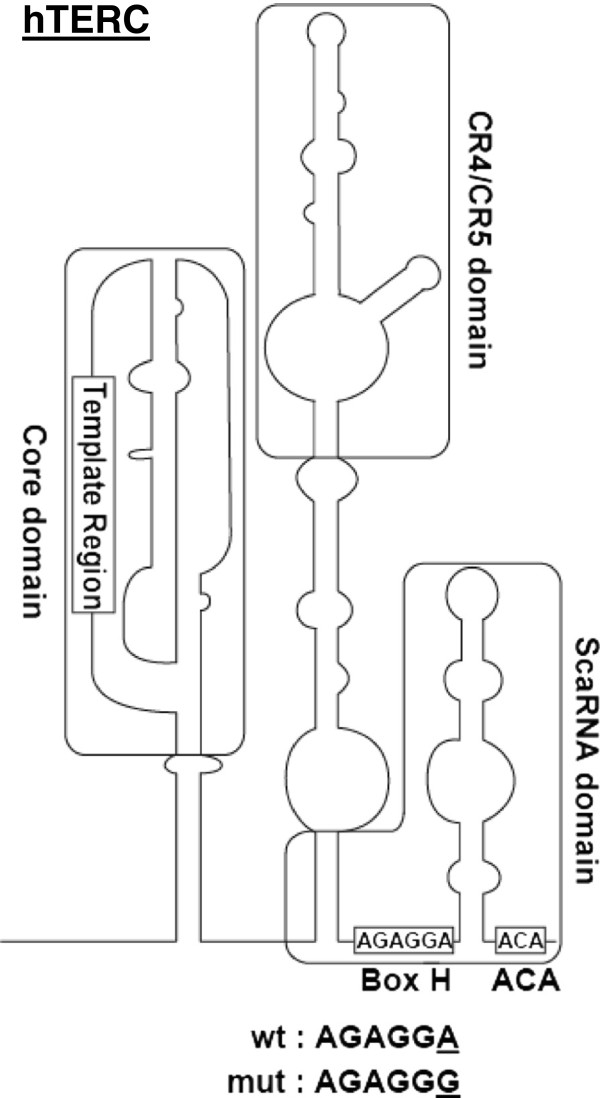
**The secondary structure of TERC showing the mutated nucleotide position identified in the patient.** The H and ACA boxes and the scaRNA domain are highlighted. The H box sequences of wtTERC and mutTERC (A377G) are illustrated, with the mutated nucleotide (A > G) shown as underlined.

TERC is an RNA component transcribed by RNA polymerase II which serves as a cognate template for telomere synthesis. In contrast to the relatively conserved TERT, TERC’s structure, size, and sequence are divergent among different species. Nonetheless, TERC’s secondary structure is similar across a wide range of eukaryotes. The human *TERC* gene (451 nucleotides (nt)) consists of three major domains: the core, the CR4/CR5, and the H/ACA scaRNA domains (Figure 1) [5]. The H/ACA scaRNA domain has two characteristic sequences, termed the H and ACA boxes, which resemble domains in small nucleolar RNAs (snoRNAs). The H/ACA boxes of *TERC* are essential for telomere biogenesis. H/ACA proteins, including dyskerin, NOP10, NHP2, and GAR1, recognize H/ACA-box sequences [6-8] and recruit TERC into a unique subnuclear organelle called Cajal body, which is crucial for assembly of telomerase components [9]. Mutations in the *TERC* gene as well as in other telomerase components cause accelerated telomere attrition, leading to disease: dyskeratosis congenita mainly in children [10-15], pulmonary fibrosis [16-18], liver cirrhosis [19], and bone marrow failure syndromes of adults (aplastic anemia, myelodysplastic syndromes (MDS), and others) [20-22].

Here, we report the association of an H-box mutation of *TERC* with human disease in a young man with bone marrow failure.

## Case presentation

### Methods

#### Patients, DNA extraction, and sequencing

A diagnosis of MDS was based on the World Health Organization (WHO) classification of myeloid neoplasms [23]. Having obtained written informed consent, peripheral blood samples were collected from our patient and his parents at the Karolinska University Hospital. Peripheral blood specimens of the patient’s parents were separated into CD3-positive and -negative populations using a magnetic-activated cell sorting (MACS) labeling system (Miltenyi Biotec, Bergishch Gladbach, Germany), according to the manufacturer’s protocols.

Peripheral blood samples of the patient, his father, son and siblings were also obtained clinically at the Clinical Genetics Laboratory, Umeå University Hospital. A sample of the patient’s sperm cells were collected before his bone marrow transplantation and analyzed for telomere length.

DNA was extracted from each separated cell population, peripheral blood specimens and the sperm sample using the QIAamp DNA Blood Mini kit or the QIAamp-96 spin blood kits (Qiagen, Hilden, Germany). DNA integrity was confirmed for all samples by agarose gel electrophoresis. All exons of the *TERT* and *TERC* genes were amplified by PCR using standard conditions and analyzed by direct sequencing using the BigDye Terminator v3.1 Cycle Sequencing kit on a ABI 3100 DNA Sequencer (Applied Biosystems, Grand Island, NY, USA), according to the manufacturer’s instruction.

#### Quantitative PCR (qPCR) analysis of telomere length

Telomere lengths of peripheral blood leukocytes in the patient and his parents were measured by qPCR as described by Cawthon [24,25], with several modifications [26], using the Qiagility robot and the Rotor-Gene Q (Qiagen). Telomere lengths of peripheral blood leukocytes in the MDS patient, his father, son and siblings, as well as of the patient’s sperm cells were also measured by PCR at Umeå University as described previously [27]. Data of telomere lengths were combined with reference to the telomere length of the patient’s peripheral blood leukocytes.

#### Single telomere elongation length analysis (STELA)

STELA was performed as previously described [28]. Briefly, PCR-amplified DNA products were resolved by 0.7% LE agarose (Ambion, Carlsbad, CA, USA) gel electrophoresis, followed by detection with a probe against telomere repeats using the *T*elo*TAGGG* Telomere Length Assay kit (Roche Applied Science, Indianapolis, IN, USA), following the manufacturer’s protocols. For each DNA specimen, five PCR reactions were performed in duplicate.

#### Plasmid construction and telomeric repeat amplification protocol (TRAP) assay

pcDNA3 TERC was constructed by inserting *TERC* sequence into pcDNA3 plasmid (Invitrogen, Carlsbad, CA, USA) at KpnI and EcoRI sites. A377G mutation was generated in pcDNA3 plasmid by using QuikChange Lightning Site-Directed Mutageneis Kit (Agilent Technologies, Santa Clara, CA, USA) according to the manufacturer’s instructions. The mutation was confirmed by Sanger sequencing. The results of the TRAP and the RNA FISH experiments were confirmed with two different A377G mutant clones.

Telomerase-deficient WI 38 VA13 subline 2RA cells (VA13; ATCC, Manassas, VA, USA) were plated on six-well culture plates (4 x 10^5^ cells/ well) and incubated for 24 hours. Either pcDNA3-wtTERC (2 μg) or pcDNA3- mutTERC (2 μg) was cotransfected with pcDNA3-Flag-TERT (2 μg) into the VA13 cells using X-tremeGENE HP DNA Transfection Reagent (12 μl, Roche Applied Science). After 48 hours, transfected cells were washed with cold PBS, and directly lysed with CHAPS buffer (EMD Millipore, Billerica, MA, USA) in the wells on ice. Protein concentration was measured with the BCA Protein Assay kit (Thermo Fisher Scientific Inc., Rockford, IL, USA), and telomerase activity was measured by the fluorescence-based TRAP assay using the TRAPeze® XL Telomerase Detection kit (EMD Millipore, Billerica, MA, USA), according to the manufacturer’s instructions.

#### RNA fluorescence in situ hybridization (RNA FISH)

pcDNA3-wtTERC or pcDNA3-mutTERC was cotransfected with pcDNA3-Flag-TERT into VA13 cells as described above, and cells were subjected to RNA FISH 48 hours after transfection as described elsewhere [29,30]. As the mutation is located at nt 377 of *TERC*, we used a mixture of three probes which do not overlap the mutation site [30,31]. All probes were aminoallyl-T-modified deoxyoligonucleotides synthesized by Integrated DNA Technologies (Coralville, IA, USA).

Probe 1) 5′-T*GCGCGCGGGGAGCAAAAGCACGGCGCCT*ACGCCCTTCTCAGTT*AGGGTTAGAC A-3′(complementary to *TERC* nt 43–96); Probe 2) 5′- GCT*GACATTTTT*TGTTTGCTCT*AGAATGAACGGT*GGAAGGCGGCAGGCCGAGGCT*T-3′(complementary to *TERC* nt 128–189); Probe 3) 5′-AT*GTGTGAGCCGAGT*CCTGGGTGCACGT*CCCACAGCTCAGGGAAT*CGCGCCGCGCT*C-3′(complementary to *TERC* nt 393–449). Aminoallyl-modified thymidines are indicated by T*. The aminoallyl-modified thymidines in the probes were chemically conjugated with Cy3 fluorophore (Cy3 monofunctional reactive dye; Amersham Pharmacia, Piscataway, NJ, USA), and the labeling efficiency was tested with Nanodrop 1000 (Thermo Scientific, Wilmington, DE, USA). Human p80-Coilin or dyskerin was detected with a mouse monoclonal anti-Coilin antibody (1:500 dilution; (Pdelta) ab11822, abcam, Cambridge, MA, USA) or a rabbit polyclonal anti-dyskerin antibody (1:50 dilution; (H-300) sc48794, Santa Cruz, Dallas, TX, USA), respectively. Slides were mounted in VECTASHIELD Mounting Medium with DAPI (Vector Laboratories, Burlingame, CA, USA). Images were acquired by confocal laser scanning microscopy with Zeiss LSM 780 confocal system (Carl Zeiss MicroImaging, NY, USA) at the Light Microscopy Core of NHLBI, NIH.

#### Medical history

**Figure 2:**
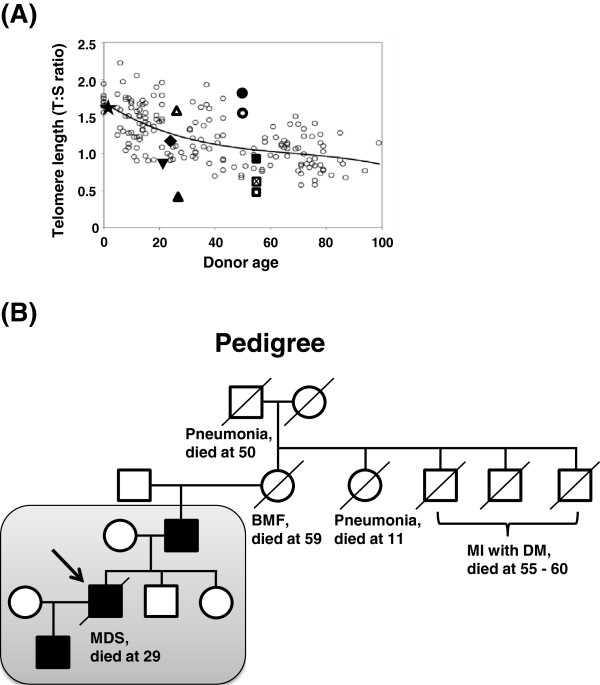
**Familial analysis of telomere length and disease manifestations. A.** The average telomere length in the patient was significantly short compared to age-matched healthy controls. Telomere length (TL) was measured by qPCR and calculated as the telomere to single copy gene ratio (T:S ratio). The figure illustrates the standard curve for age-related telomere length shortening in healthy donors (small open circles). The TL of each specimen is shown as follows; the patient peripheral blood (PB), ▲; the patient’s sperm, Δ; PB of the patient’s son, ★; whole PB of the patient’s father, ☒, CD3-negative PB of the patient’s father, ■; CD3-positive PB of the patient’s father, □; CD3-negative PB of the patient’s mother, ●; CD3-positive PB of the patient’s mother, ○; and PB of the patient’s siblings, sister ▼and brother ◆. **B.** Pedigree analysis. The family members within the shaded box were tested for the A377G *TERC* mutation and telomere length. The proband is indicated with an arrow. Filled symbols represent individuals carrying the A377G mutation, whereas open symbols are individuals with no mutation. Squares; males, and circles; females. BMF, bone marrow failure; MI, myocardial infarction; DM, diabetes mellitus; and MDS, myelodysplastic syndrome.

The patient, born in 1983, was diagnosed with idiopathic thrombocytopenic purpura (ITP) in 1994, but no clinical follow-up was performed. In 2007, he was diagnosed with hypoplastic MDS with platelets 25 x 10^9^/l. Cytogenetic analysis in 2008 showed a partial trisomy 1q with 46,XY,der(13)t(1;13)(q11;p13) in 11 of 25 metaphase cells. In 2009, the patient was referred to the Karolinska University Hospital with pancytopenia showing leukocytes 2.0 x 10^9^/l, hemoglobin 10.0 g/dl, and platelets 20 x 10^9^/l. Absence of cytogenetic abnormalities and markedly reduced marrow cellularity on subsequent bone marrow biopsies (in 2009 and 2010) resulted in revision of the diagnosis from MDS to aplastic anemia (AA). The patient became transfusion-dependent (red blood cells and platelets) and susceptible to infection due to severe neutropenia (leukocyte < 0.2 x 10^9^/l). He underwent bone marrow transplantation in 2012, but died of Epstein-Barr virus-associated B-cell lymphoma in the same year. The patient’s parents and two younger siblings were healthy, but the paternal grandmother had died with bone marrow hypoplasia at age 59. The grandmother’s sister died of pneumonia at age 11, and her father died of pneumonia at age 50 (Figure 2B).

#### Molecular analysis

DNA samples of the patient and his parents were sent to the National Heart, Lung and Blood Institute for analysis in 2011. Since we suspected that the pedigree carried a genetic mutation in a telomerase-associated gene, we examined telomere content by qPCR. The telomere length of white blood cells in the patient was extremely short as compared to age-matched healthy controls (Figure 2A). Analysis of the relatives showed that the father had shorter telomere length than the controls, whereas both siblings and the patient’s son showed normal-for-age telomere content.

**Figure 3:**
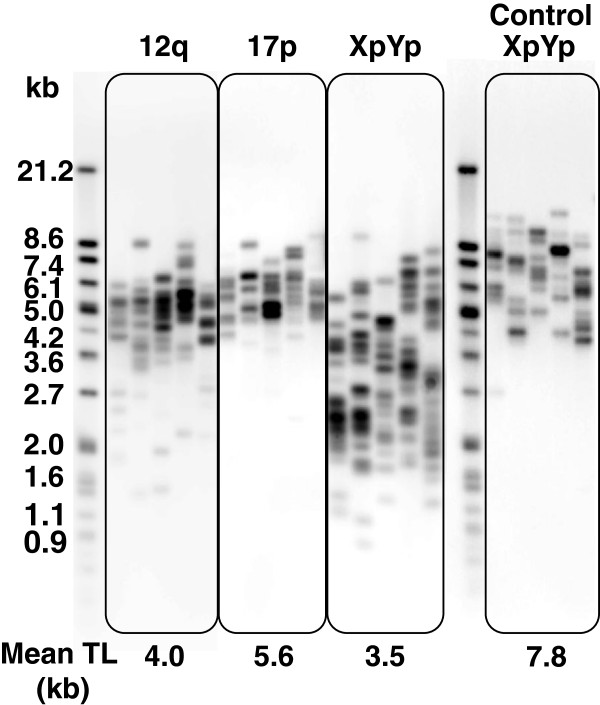
**STELA showed extremely short telomeres in the patient.** STELA was employed to address telomere length and distribution in the patient in a chromosome-specific manner (chromosomes XpYp, 12q, and 17p). A representative XpYp STELA obtained from a healthy donor (age 38 years) is shown as a control. Mean telomere length (TL; kb) is represented under each STELA result. Molecular weight markers (kb) are indicated on the left.

To further examine telomere attrition in our patient, we employed the PCR-based technique STELA for measurement of single telomere lengths in a chromosome-specific manner. Southern blot visualization of STELA was obtained by using telomere-specific probes (Figure 3). Telomere lengths in 12q and especially in XpYp were widely distributed with many short telomeres, whereas they were less heterogeneous in 17p (Figure 3). Telomere lengths in controls were well-maintained within a defined length range, compared to those of the patient. 17p telomere length in controls also displayed low variation (data not shown); 17p telomere length distributions have been reported to be less heterogeneous compared to other chromosomes in fibroblasts and peripheral blood leukocytes [32].

As the patient had very short telomeres, we sequenced all exons of the *TERT* and *TERC* genes. Only a single heterozygous mutation at nt 377 (A377G) of *TERC* was identified within the H box of the H/ACA scaRNA domain (Figure 1). The same mutation was also found in the patient’s father, both in CD3-positive and CD3-negative populations, but not in the mother, suggesting origin in the paternal germline. An extended family analysis also identified the A377G mutation in the patient’s son, whereas the patient’s two healthy siblings did not have the mutation. A paternal influence has been reported on telomere length of offspring [33,34], but the patient’s son showed normal telomere length despite his A377G mutation (Figure 2A). To clarify the inheritance of short telomere length of the patient, we examined the telomere length of the patient’s sperm cells: surprisingly, the telomere length of the sperm cells was much longer compared to the patients’ leukocytes (Figure 2A).

**Figure 4:**
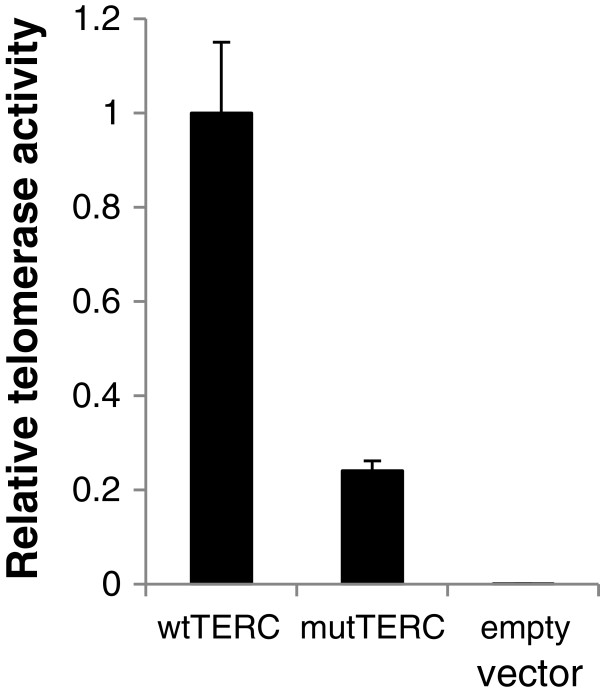
**TRAP assay showed significantly low telomerase activity in the mutTERC (A377G).** VA13 cells were co-transfected with either pcDNA3-wtTERC or pcDNA3-mutTERC together with pcDNA3-Flag-TERT, followed by telomerase activity measurement. Results were calculated as a relative activity compared to values obtained from pcDNA3-wtTERC and pcDNA3-Flag-TERT. Bars denote standard deviations. A representative result is shown here.

To address whether the *TERC* A377G mutation was responsible for telomere shortening in the patient, telomerase activity was measured by a TRAP assay, in which pcDNA3-wtTERC or mutTERC was co-transfected with pcDNA3-Flag-TERT into telomerase-deficient VA13 cells. Telomerase activity was significantly reduced (70 – 90%) in VA13 cells that had been transfected with the mutant *TERC* plasmid (Figure 4).

**Figure 5:**
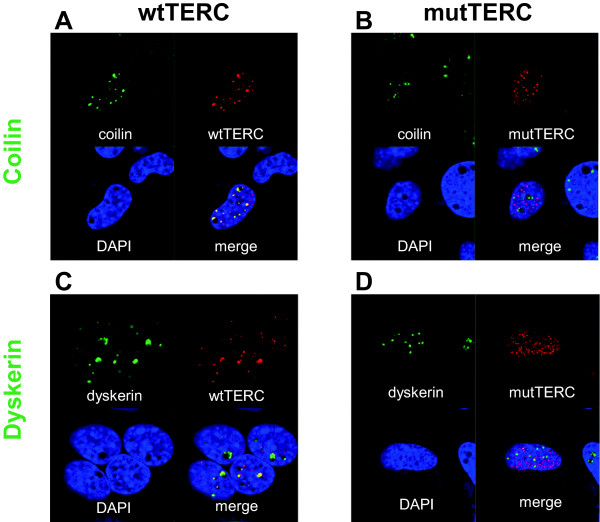
**mutTERC did not colocalize with dyskerin or Cajal bodies.** Telomerase-defective VA13 cells were transfected with either pcDNA3-wtTERC (**A** and **C**) or pcDNA3-mutTERC (**B** and **D**) together with pcDNA3-Flag-TERT and subjected to RNA FISH combined with immunofluorescence. wtTERC or mutTERC was labeled with Cy3-conjugated probes (red, **A-D**), Cajal bodies and dyskerin were stained with anti-Coilin antibody (green, **A** and **B**) and anti-dyskerin antibody (green, **C** and **D**), respectively. Nucleus was stained with DAPI (blue, **A-D**).

Mutations in the H/ACA boxes of the *TERC* gene have been reported to be important for TERC to assemble with H/ACA proteins including dyskerin [35]. To test the hypothesis that the *TERC* A377G mutation might cause decreased telomerase activity through impaired interaction of TERC with H/ACA proteins or mislocalization of TERC in the nucleus, we performed RNA FISH using VA13 cells transiently transfected with pcDNA3-wtTERC /mutTERC. mutTERC did not colocalize with Cajal body marker coilin (Figure 5A and B). Furthermore, mutTERC did not colocalize with dyskerin (Figure 5C and D).

### Discussion

In this study, we have identified a mutation A377G (AGAGGA → AGAGGG) in the H box of *TERC* in a young patient with bone marrow failure. The patient displayed short telomeres and significantly reduced telomerase activity *in vitro* in the TRAP assay, suggesting that the H box of TERC is crucial for telomere biogenesis in humans. Analysis of the pedigree showed that the mutation was germline. The father had no clinical symptoms, by the age of 58, although he carried the mutation, consistent with previous reports which have observed variable penetrance of disease manifestations in patients carrying *TERC* mutations [21,36]. The patient’s son carrying the mutation had also not shown clinical symptoms and he had normal telomere length at age one year. However, close follow-up of the boy in the future would be necessary.

The A377G *TERC* mutation was previously reported in an aplastic anemia patient, but no functional assay of the mutation was performed [37]. The H box is extensively conserved among snoRNAs and scaRNAs in eukaryotes, sharing the consensus sequence “ANANNA (A: adenine, N: any nucleotide)”. Microinjection experiments in *Xenopus* oocytes have shown that all three conserved adenine nucloetides of the H box are essential for nucleolar localization of snoRNA U64 [38]. A human TERC mutant, which was created by replacement of all adenines with uracil in the conserved H box residues (AGAGGA → UGUGGU), was undetectable in transiently transfected 293 cells using polyacrylamide gel electrophoresis [6]. Another H-box mutant (A372U) was subjected to *in vitro* RNA synthesis and immunoprecipitation experiments, showing severely impaired ribonucleoprotein (RNP) formation and a critical role of the H box for assembly of hTERC with H/ACA proteins [35]. These studies demonstrate that integrity of the H box is essential for hTERC stability, intracellular trafficking, and assembly of hTERC with H/ACA proteins.

In our study, RNA FISH experiments showed that mutTERC did not accumulate in Cajal bodies, and did not colocalize with dyskerin. Mislocalization of mutTERC with dyskerin is consistent with the previous report [35]. As dyskerin has been reported to be necessary for the active telomerase enzyme complex [39], lack of association of mutTERC with dyskerin might have caused shorter-for-age telomere length in our patient. mutTERC did not accumulate in Cajal bodies in the absence of CAB box mutations. Lack of pre-RNP formation might have caused this mislocalization. Ideally, telomerase activity should have been tested on the patient’s samples, but these were not available.

Of interest, telomere content of the patient’s sperm cells was much greater compared to his leukocytes. Sperm telomere length has been reported to correlate with that of white blood cells in the same individual [40], but sperm telomere length also has been reported to increase with age [40-43]. The mechanism of elongation is not understood, but upregulation of *hTERT* is suggested to contribute [44-46]. Upregulation of *hTERT* might have overcome a decrease of telomerase activity due to the A377G *TERC* mutation in our patient. A decrease in telomerase activity with telomerase mutations leads to telomere shortening with clinical manifestations in the hematopoietic system, but might not occur during spermatogenesis. Disease anticipation has been reported in *TERC* RNA haploinsufficiency [36,47]. Azospermia was observed in one affected individual, suggesting that spermatogenesis was also affected by *TERC* deletion [47]. Our case suggests that severity of telomere shortening during spermatogenesis might be different depending on mutation sites of *TERC*. Further analysis of individuals with telomerase mutations is needed to examine to what extent telomere length maintenance in sperm and bone marrow differs.

## Conclusions

The A377G mutation was identified in the H box of *TERC* in a young MDS patient with a chromosomal abnormality. As telomeres protect chromosomes from instability, it is highly plausible that this genetic lesion was responsible for the patient’s hematological manifestations, including marrow failure and aneuploidy in the hematopoietic stem cell compartment.

### Consent

The patient and his parents provided written informed consent for genetic testing, and publication of the results following the protocol approved by the institutional review board of the National Heart, Lung, and Blood Institute, protocol 04-H-0012 (http://www.ClinicalTrials.gov identifier: NCT00071045). Analyses of peripheral blood samples from the patient’s son and siblings were performed clinically after informed consent. Sperm DNA was analyzed after approval by the Ethical Committee at Umeå University (#2013/157-31).

## Abbreviations

TERC: Telomerase RNA component; MDS: Myelodysplastic syndrome; TERT: Telomerase reverse transcriptase; TRAP: Telomeric repeat amplification protocol; STELA: Single telomere elongation length analysis; RNA FISH: RNA fluorescence in situ hybridization.

## Competing interests

The authors declare that they have no competing interests.

## Authors’ contributions

YU and SK carried out the molecular genetic studies, designed the study, and drafted the manuscript. RTC carried out the sequencing analysis, qPCR analysis, and designed the study. AN and GR performed sequencing and qPCR analysis (see Methods) and drafted the manuscript. EHL performed DNA extraction and provided vital clinical samples and information. NSY coordinated and supervised the study. All the authors revised and approved the final version of the manuscript.

## Pre-publication history

The pre-publication history for this paper can be accessed here:

http://www.biomedcentral.com/1471-2350/15/68/prepub
